# Mitochondrial Generated Redox Stress Differently Affects the Endoplasmic Reticulum of Circulating Lymphocytes and Monocytes in Treatment-Naïve Hodgkin’s Lymphoma

**DOI:** 10.3390/antiox11040762

**Published:** 2022-04-11

**Authors:** Cecilia Marini, Vanessa Cossu, Matteo Bauckneht, Sonia Carta, Francesco Lanfranchi, Francesca D’Amico, Silvia Ravera, Anna Maria Orengo, Chiara Ghiggi, Filippo Ballerini, Paolo Durando, Sabrina Chiesa, Alberto Miceli, Maria Isabella Donegani, Silvia Morbelli, Silvia Bruno, Gianmario Sambuceti

**Affiliations:** 1 CNR, Institute of Molecular Bioimaging and Physiology (IBFM), 20054 Milan, Italy; 2 IRCCS, Ospedale Policlinico San Martino, 16132 Genoa, Italy; vane.6291@gmail.com (V.C.); matteo.bauckneht@hsanmartino.it (M.B.); sonia.carta@hsanmartino.it (S.C.); annamaria.orengo@hsanmartino.it (A.M.O.); chiara.ghiggi@hsanmartino.it (C.G.); filippo.ballerini@hsanmartino.it (F.B.); paolo.durando@unige.it (P.D.); sabrina.chiesa@hsanmartino.it (S.C.); silviadaniela.morbelli@hsanmartino.it (S.M.); sambuceti@unige.it (G.S.); 3 Department of Health Sciences, University of Genoa, 16132 Genoa, Italy; dr.francescolanfranchi@gmail.com (F.L.); damicofrancesca@houtlook.com (F.D.); albertomiceli23@gmail.com (A.M.); isabella.donegani@gmail.com (M.I.D.); 4 Department of Experimental Medicine, Human Anatomy, University of Genoa, 16132 Genoa, Italy; silvia.ravera@unige.it (S.R.); silvia.bruno@unige.it (S.B.); 5 Department of Internal Medicine, University of Genoa, 16132 Genoa, Italy

**Keywords:** cancer, lymphoma, redox stress, 2-NBDG, mitochondria, endoplasmic reticulum, pentose phosphate pathway

## Abstract

Background. The redox stress caused by Hodgkin’s lymphoma (HL) also involves the peripheral blood mononucleated cells (PBMCs) even before chemotherapy. Here, we tested whether lymphocytes and monocytes show a different response to the increased mitochondrial generation of reactive oxygen species (ROS). Methods. PBMCs, isolated from the blood of treatment-naïve HL patients and control subjects, underwent assessment of malondialdehyde content and enzymatic activity of both hexose- and glucose-6P dehydrogenase (H6PD and G6PD) as well as flow cytometric analysis of mitochondrial ROS content. These data were complemented by evaluating the uptake of the fluorescent glucose analogue 2-NBDG that is selectively stored within the endoplasmic reticulum (ER). Results. Malondialdehyde content was increased in the whole population of HL PBMCs. The oxidative damage matched an increased activity of G6PD, and even more of H6PD, that trigger the cytosolic and ER pentose phosphate pathways, respectively. At flow cytometry, the number of recovered viable cells was selectively decreased in HL lymphocytes that also showed a more pronounced increase in mitochondrial ROS generation and 2-NBDG uptake, with respect to monocytes. Conclusions. PBMCs of HL patients display a selective mitochondrial and ER redox stress most evident in lymphocytes already before the exposure to chemotherapy toxicity.

## 1. Introduction

Among the different host–cancer interactions able to modulate disease aggressiveness, the inflammatory response seems to represent a double-edged sword, mostly because of the heterogeneous composition of inflammatory infiltrates. Indeed, the secretion of trophic factors by tumor-associated macrophages contributes to neovascularization and lesion growth [1,2,3,4], as opposed by the immunosurveillance warranted by a high ratio of cytolytic/regulatory T lymphocytes. Tumor-associated macrophages are derived from peripheral blood monocytes whose number predicts a poor outcome in several cancer types, especially in Hodgkin’s lymphoma (HL) [5,6]. By contrast, lymphocyte count represents a surrogate of host immune competence and is thus directly correlated with survival [7,8]. These considerations thus suggest that the inflammatory responses triggered by HL lesions extend to contaminating the peripheral blood mononucleated cells (PBMCs), despite the substantial absence of neoplastic elements in the bloodstream [9,10,11].

According to the wide literature, inflammatory response implies a metabolic switch characterized by a simultaneous increase in glycolytic flux and oxygen consumption rate (OCR) fueling the generation of the large amounts of reactive oxygen species (ROS) needed for the activation of involved immune cells [12,13,14]. This metabolic adaptation largely involves the dynamic interactions between the mitochondria and the endoplasmic reticulum (ER) through the mitochondria-associated membranes (MAMs) [15,16]. On one side, these interfaces represent a critical site for inflammasome formation [15,17,18]. On the other hand, they also configure the ER as a preferential target for the accelerated mitochondrial generation of reactive oxygen species (ROS) [19,20]. This latter consideration suggests the presence of a local metabolic response to the enhanced reticular redox stress.

To the best of our knowledge, this hypothesis has not been tested so far. Nevertheless, it agrees with the notion that the ER is enriched with a complete enzymatic asset to activate an autonomous pentose phosphate pathway (PPP) [21,22,23]. This pathway is triggered by the enzyme hexose-6P-dehydrogenase (H6PD) that, differently from its cytosolic counterpart glucose-6P-dehydrogenase (G6PD), can process a large variety of hexoses. In agreement with this concept, we recently reported that the activation of ER-PPP is the main determinant of the high uptake of two glucose analogues conventionally used to estimate glycolytic flux, namely the PET tracer ^18^F-fluoro-deoxyglucose and the fluorescent probe 2-(N-(7-nitrobenz-2-oxa-1,3- diazol-4-yl)-amino)-2-deoxyglucose (2-NBDG)) [24,25,26,27].

Accordingly, the present study was designed to verify the hypothesis that the PBMCs of HL patients display the oxidant phenotype typical of inflammatory activation modifying the mitochondria–ER redox balance before the start of chemotherapy, and whether lymphocytes and monocytes display a divergent susceptibility to the redox stress.

## 2. Materials and Methods

### 2.1. Controls and Patients

The study included 19 patients admitted to our institute for suspected, and subsequently confirmed, HL. Control subjects (*n* = 62) were enrolled from normalcy database collected from our institute in the Preventive Medicine Program. Exclusion criteria were positivity for HBV, HCV and HIV or any other coexistent disease asking for pharmacological therapy. All participants provided their written informed consent to participate in this study that was approved by the Ethical Committee of Regione Liguria (50/20—DB id 10306).

Blood samples of HL and control subjects were collected from January 2020 to June 2021. Blood cell composition was characterized according to the same routine procedure of our institute for both cohorts, before chemotherapy start in HL patients, and at the scheduled time in control subjects.

### 2.2. PBMCs Isolations

At the time of blood sampling, further 15 mL of blood was collected and transferred into our laboratory to be analyzed within 24 h. According to standard procedures [28,29], PBMCs were isolated using lympholyte gradients (Cedarlane), washed three times with Ca^2+/^Mg^2+^ free phosphate-buffered saline (PBS) and resuspended at 5 × 10^6^ cells/mL. Obtained cells were divided and dedicated to the different experimental evaluations.

### 2.3. Flow Cytometric Analysis

In the first set, 5 × 10^5^ PBMCs of either HL patients or controls were stained for 20 min at 37 °C with 5 μM MitoSOX Red, 1 μM ER-Tracker Red or 50 μM 2-NBDG (all from Invitrogen by Thermo Fisher Scientific, Waltham, MA, USA). The cells were then washed two times with PBS, centrifuged for 5 min at 290× *g* and resuspended in PBS + 1% BSA for flow cytometric analysis. Data were acquired on a FACSCan (Becton Dickinson, Milan, Italy) and data analysis was performed with FlowJo software. The analysis was restricted to viable PBMCs, after gating procedures based on forward- and side-scatter features [30]. In a subset of five control subjects and eight HL patients, the recovery of lymphocytes and monocytes in the expected gate was confirmed by staining with CD3, CD19 and CD14 (Miltenyi Biotec, Bergisch Gladbach, Germany). Cellular viability was evaluated by propidium iodide (PI) exclusion assays. Cells were stained with 1 μg/mL PI (Enzo Life Sciences, Milan, Italy) and PI fluorescence measured after 5 min.

### 2.4. Seahorse Analysis

For this second set of experiments, 10^5^ PBMCs/well were seeded in XFp cell plates and centrifuged gently with no brake at 40× *g* for 3 min, and the plate then rotated 180° before centrifugation again at 80× *g* for 3 min to encourage adhesion to the plate and the forming of an evenly dispersed monolayer [31,32]. OCR and extracellular acidification rate (ECAR) were determined using the Seahorse XFp Extracellular Flux Analyzer (Agilent Technologies, Santa Clara, CA, USA). Cells were then incubated at 37 °C for 45 min in no-CO_2_ incubator with Agilent Seahorse DMEM, pH 7.4, enriched with glucose (11 mM). OCR and ECAR were monitored according to the manufacturer instructions. Briefly, three measurements of OCR and ECAR were taken under control conditions and after sequential injections of 1.5 μM oligomycin (ATP synthase inhibitor) and 0.5 μM rotenone (Complex I inhibitor) plus 0.5 μM antimycin A (Complex III inhibitor).

### 2.5. Enzymatic Assays

For the third set of experiments, PBMCs were again centrifuged at 300× *g* for five minutes and were suspended in PBS supplemented by protease inhibitors to be sonicated twice for 10 s in ice, with a break of 30 s. The activity of H6PD and G6PD were assayed using an absorbance microplate reader (ELx808™, Winooski, VT, USA) to follow the reduction of NADP at 340 nm [24,25,26,33]. H6PD enzymatic function was tested in the presence of Tris-HCl pH 7.4 100 mM, glucose 10 mM, and NADP 0.5 mM. By contrast, G6PD activity was assayed in the presence of Tris-HCl pH 7.4 100 mM, glucose-6-phosphate (G6P) 10 mM, and NADP 0.5 mM.

Malondialdehyde (MDA) levels were evaluated, by the thio-barbituric acid reactive substances assay, using a UV/visible spectrophotometer (Ultraspec 2000, Pharmacia Biotech, Erie, PA, USA) [26,34]. In all cases, enzymatic activity was normalized for total protein concentrations tested using Bradford analysis [35].

Finally, total antioxidant capacity was evaluated following the manufacturer’s instructions of a dedicated kit (MAK187, Sigma, St. Louis, MO, USA) that provides a complete description of the total cell antioxidant power associated with the endogenous scavengers, expressed as Trolox equivalent antioxidant capacity content.

### 2.6. Statistics

All data were reported as means of ± standard deviation. Unpaired t test was used to compare data in different groups. Univariate linear regression analysis was performed by using the least squares method. A *p* < 0.05 was considered statistically significant. All statistical analyses were carried out by using dedicated software packages, namely SPSS, v20 (SPSS, Chicago, IL, USA) and GraphPad Prism 9.3.1 (GraphPad, San Diego, CA, USA).

## 3. Results

### 3.1. Patient Population

As shown in Table 1, control subjects and HL patients showed comparable age and similar prevalence of female gender. At routine evaluation of peripheral blood, red blood cell count and hemoglobin assay reported similar values in the two groups. However, HL patients showed a significant increase in white blood cell number that mostly involved both neutrophils and monocytes, while lymphocyte counts were comparable in both groups.

### 3.2. Redox Stress in PBMCs

**Figure 1:**
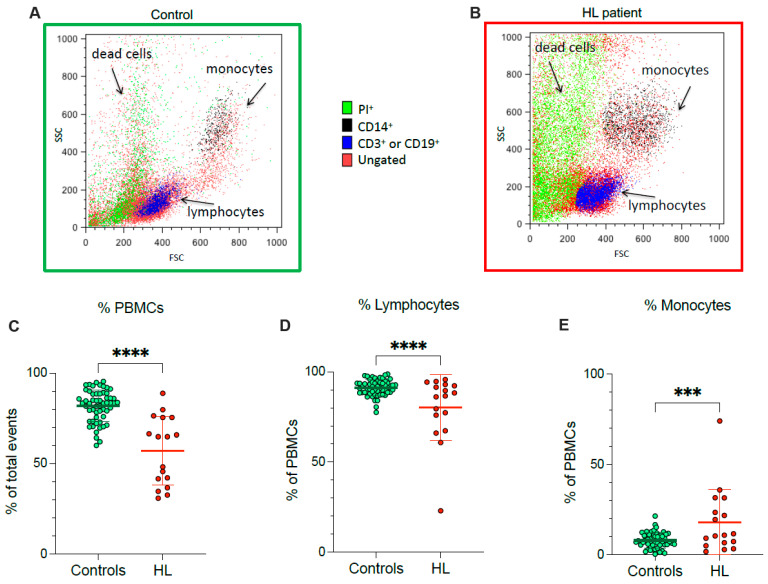
*PBMC cell culture.* Representative flow cytometry dot plots of forward- and side-scatter features (FSC, x-axis, and SSC, y-axis) of PBMCs showing the recovery of lymphocytes and monocytes in the expected gates of controls (**A**) and HL patients (**B**). Percentage of PBMCs (**C**), lymphocytes (**D**) and monocytes (**E**) sampled from controls (green) and HL patients (red). Graphs display individual data and mean ± SD. *** = *p* < 0.002, **** = *p* < 0.001.

According to flow cytometric analysis, HL patients showed a lower fraction of recovered viable PBMCs, and a concomitant rise in dead cell fraction, with respect to control subjects (Figure 1A–C). In agreement with the different PBMC counts documented at routine evaluation, the decrease in recovered viable cells was particularly evident for lymphocytes (Figure 1D), while the viable monocyte fraction remained higher in HL samples than in control ones (Figure 1E).

**Figure 2:**
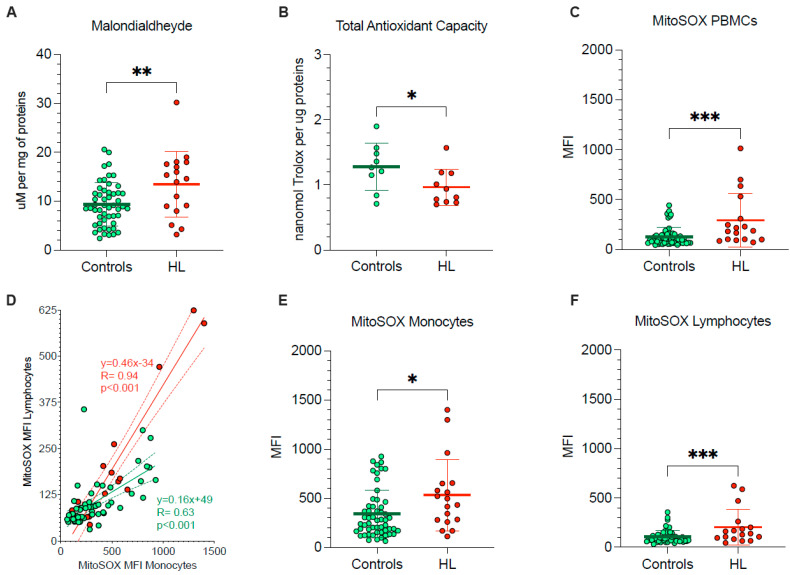
*PBMCs oxidative stress and antioxidant response.* (**A**) Malondialdehyde content and (**B**) total antioxidant capacity evaluated in lysed PBMCs. (**C**) Mean fluorescence intensity (MFI) of MitoSOX evaluated in all PBMCs of controls (green) and HL patients (red). (**D**) Correlation between MitoSOX MFI in monocytes (x-axis) and lymphocytes (y-axis) of controls (green) and HL patients (red). Simple linear regression (continuous line) and 95% confidence bands of the best-fit line (dashed line) of controls (green) and HL patients (red). MitoSOX signal measured as mean fluorescence intensity (MFI) in lymphocytes (**E**) and in monocytes (**F**) of controls (green) and HL patients (red). Graphs display individual data and mean ± SD. * = *p* < 0.05, ** = *p* < 0.01, *** = *p* < 0.009.

The pronounced vulnerability of HL PBMCs agreed with the occurrence of oxidative damage, documented by the significant enhancement in their MDA content (Figure 2A). By contrast, total antioxidant capacity was decreased in HL PBMCs with respect to control cells (Figure 2B), in the sub-group of studied HL patients and control subjects.

To explain the redox stress of HL PBMCs, we focused our attention on mitochondrial levels of superoxide radical (O^2−^) (Figure 2C–F). MitoSOX Red signal in lymphocytes and monocytes was directly correlated in both HL patients and control subjects indicate that factors influencing mitochondrial ROS production were at least partially shared by the two PBMC types (Figure 2D). Nevertheless, the slope of the identified regression line was significantly (*p* < 0.001) steeper in HL than in the control subjects (Figure 2D), suggesting a higher susceptibility to HL effect by mitochondria populating lymphocytes with respect to monocytes. As a consequence, HL lymphocytes showed a 98% increase in MitoSOX fluorescence, as opposed to the 53% rise observed in monocytes with respect to corresponding control samples, eventually resulting in a higher probe uptake when the whole PBMC population was analyzed (Figure 2C–F).

Therefore, PBMCs harvested from treatment-naïve HL patients showed an evident oxidative damage, as documented by the increased level of the final product of polyunsaturated fatty acid peroxidation, i.e., MDA. This same damage, in turn, largely involved mitochondrial ROS generation and was more pronounced in lymphocytes than in monocytes.

### 3.3. Evaluation of Oxidative Phosphorylation

**Figure 3:**
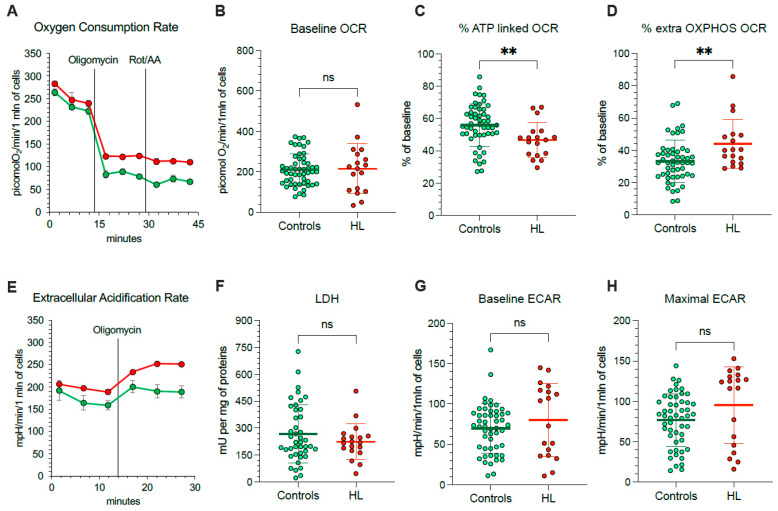
*Mitochondrial energetic function in PBMCs.* (**A**) Typical trace from the Seahorse XFp of oxygen consumption rate (OCR) over time (x-axis) measured on intact PBMCs (10 × 10^5^ cells/well) collected from controls (green) and HL patients (red). Injections of ATP-synthase inhibitor Oligomycin A, and rotenone/antimycin A (Rot/AA) are indicated with black lines. Average and SD of at least 3 replicate wells are plotted. (**B**) Basal OCR measured in presence of glucose (11 mM) and (**C**) after injection of Oligomycin A expressed as % of corresponding basal OCR. (**D**) Extramitochondrial OCR after the inhibition of Complexes I, III, and V after sequential injection of Oligomycin A and Rot/AA, expressed as % of corresponding basal OCR. (**E**) Typical trace from the Seahorse XFp of extracellular acidification rate (ECAR) over time (x-axis) measured on intact PBMCs (10 × 10^5^ cells/well) collected from controls (green) and HL patients (red). Injections of Oligomycin A is indicated with black lines. Average and SD of at least 3 replicate wells are plotted. (**F**) Lactate dehydrogenase (LDH) activity, (**G**) basal ECAR measured in presence of glucose (11 mM) and (**H**) maximal ECAR after injection of Oligomycin A. Graphs display individual data and mean ± SD. ** = *p* < 0.01.

Considering that mitochondria represent, at the same time, a main ROS source and a primary target of ROS-induced damage, we aimed to understand the mechanisms underlying their selective redox stress. We thus extended our evaluation to the respiratory function (Figure 3A) and glycolytic flux of PBMCs, using the Seahorse technology. OCR was similar in HL and normal cells under control condition (Figure 3B).

Nevertheless, OCR response to ATP-synthase inhibition by oligomycin was lower in HL than in control PBMCs (Figure 3C), indicating that a smaller OCR fraction was dedicated to ATP production in HL patients with respect to control subjects. The presence of a relative respiratory uncoupling was also associated with an increase in mitochondria-independent OCR, evaluated after simultaneous inhibition of Complexes I, III and V by rotenone/antimycin (Rot/AA) and oligomycin, respectively. Indeed, the OXPHOS-independent OCR was higher in HL PBMCs with respect to control ones (Figure 3D).

The non-energetic nature of OCR in HL PBMCs was confirmed by the analysis of glycolytic flux (Figure 3E). In agreement with the invariance of LDH activity (Figure 3F), baseline ECAR was only slightly, and not significantly, increased in patients with respect to controls (Figure 3G). More importantly, this same observation also applied to maximal glycolytic capacity, estimated by ECAR under oligomycin incubation, that was remarkably similar in both cohorts (Figure 3H).

### 3.4. ER Response to OXPHOS Uncoupling in HL PBMCs

**Figure 4:**
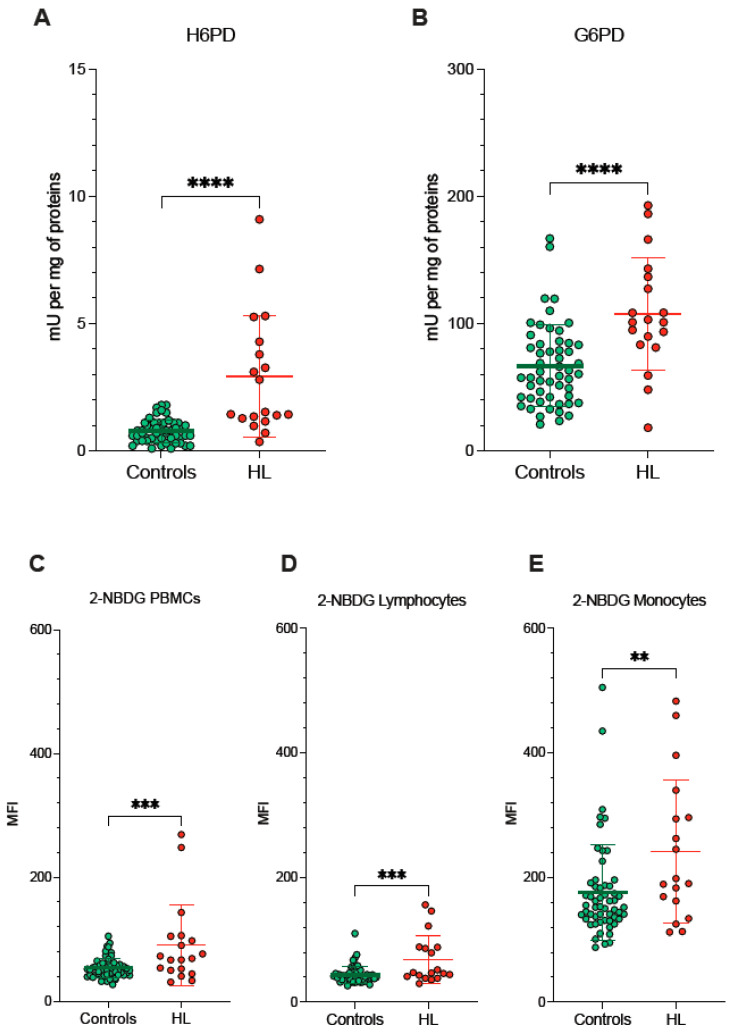
*ER- and cytosolic- PPP activity.* Catalytic function of H6PD (**A**) and G6PD (**B**), measured by enzymatic assay in lysed PBMCs sampled from controls (green) and HL patients (red). 2-NBDG uptake measured as mean fluorescence intensity (MFI) in all PBMCs (**C**), in lymphocytes (**D**) and in monocytes (**E**) of controls (green) and HL patients (red). Graphs display individual data and mean ± SD. ** = *p* < 0.01, *** = *p* < 0.002, **** = *p* < 0.001.

Since redox environments in ER and mitochondria are strictly interconnected by the exchange of metabolites and cofactors through the MAMs, we evaluated the activity of pathways dedicated to the oxidative balance in the ER lumen and in the connected MAMs. ER-PPP was activated in HL PBMCs as documented by the fourfold increase in the catalytic function of its triggering enzyme H6PD with respect to the control PBMCs (Figure 4A). The response of the cytosolic PPP was markedly less pronounced, since the activity of its triggering enzyme G6PD approximately doubled in blood cells sampled from HL patients (Figure 4B).

**Figure 5:**
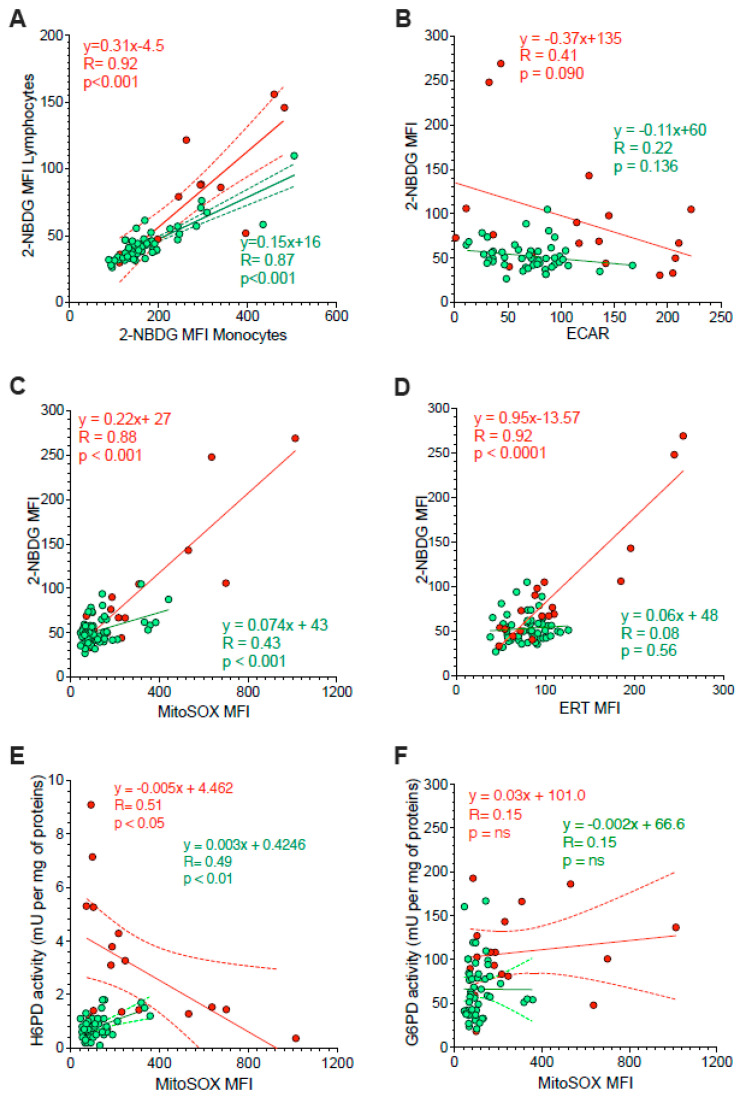
*Correlation between ER function and glycolysis or mitochondria redox stress.* Panel (**A**) displays the correlation between 2-NBDG MFI of monocytes (x-axis) and lymphocytes (y-axis) sampled from controls (green) and HL patients (red). Simple linear regression (continuous line) and 95% confidence bands of the best-fit line (dashed line) of controls (green) and HL patients (red). Shifting to the analysis of the whole PBMC population, panel (**B**) displays the absent proportionality between 2-NBDG and extracellular acidification rate (ECAR). By contrast, panel (**C**) reports the direct relation between 2-NBDG and MitoSOX fluorescence. The role of ER in 2-NBDG uptake is confirmed in panel (**D**) by the direct correlations between the fluorescence of this glucose analogue and that of the ERTracker glibenclamide (ERT). The relevance of ER-PPP in the response to the increase in mitochondrial ROS generation is indicated by the inverse correlation between MitoSOX fluorescence (*x*-axis) and H6PD activity (*y*-axis) reported in panel (**E**). This same correlation did not involve G6PD activity (**F**). Continuous lines represent the identified function while dashed lines represent the 95% confidence bands of the best-fit in both controls (green) and HL patients (red).

Besides the divergent intracellular location, the main difference between G6PD and H6PD relies on the fact that the former only recognizes glucose-6P, while the latter can process many hexoses including 2-NBDG, whose cell retention is thus linked to the activation of ER-PPP [24,25,26,27,33,34]. In agreement with this consideration, the analysis of 2-NBDG fluorescence provided results comparable to the data provided by MitoSOX Red (Figure 4C–E). On one side, probe uptake values in lymphocytes and monocytes were directly and strictly correlated in both HL patients and control subjects (Figure 5A). On the other side, the slope of the identified regression line was significantly (*p* < 0.001) steeper in HL than in control subjects (Figure 5A). The metabolic response of lymphocytes to the selective HL-related increase in mitochondrial ROS generation was thus associated with a selective activation of H6PD catalytic function.

The link between 2-NBDG uptake and ER-PPP activation was further confirmed by several observations. On one side, probe fluorescence was largely independent of the glycolytic flux estimated by ECAR in the whole PBMC populations of both HL patients and control subjects (Figure 5B). By contrast, 2-NBDG uptake was directly correlated with the fluorescence of both MitoSOX Red and the reticular probe glibenclamide (Figure 5C,D, respectively). Both correlations were well evident in HL patients, although the limited intensities of the two signals prevented the identification of this relationship in control samples (Figure 5B).

The crucial role for ER-PPP in the response to the mitochondrial ROS generation was confirmed by the inverse correlation between MitoSOX fluorescence and H6PD function (Figure 5E), observed in HL PBMCs. By contrast, this inverse relation did not involve G6PD catalytic function (Figure 5F). This finding suggests a preferential response of the ER-PPP in counterbalancing the redox stress caused by OXPHOS uncoupling with respect to its cytosolic counterpart.

According to these data, we used a univariate regression analysis aiming to verify which of the interrogated variables indeed predicted the uptake of 2-NBDG in the PBMCs. As reported in Table 2, this fluorescence intensity was predicted by all descriptors of redox stress and oxidative damage, including the presence of HL and the tolerance to the procedure used for flow cytometric analysis.

## 4. Discussion

The major finding of the present study is the evidence of oxidative damage in the PBMCs of treatment-naïve HL patients, strictly related to an accelerated ROS generation by mitochondria. The OXPHOS uncoupling and the consequent redox stress more severely hampers lymphocytes than monocytes. In both cell types, the metabolic response to the respiratory impairment implies a marked enhancement of H6PD activity and thus of glucose flux through ER-PPP. This metabolic alteration already occurs before the treatment start and should thus be considered in studies focusing on chemotherapy toxicity.

The observation of a high MDA content in HL PBMCs agrees with previous studies documenting high levels of lipid peroxidation products in serum [36] and platelets [37] as well as an accelerated ROS generation in monocytes [38] harvested from HL patients. Methodological limitations did not permit differentiating the severity of oxidative damage in the two studied cell types. Nevertheless, the high mitochondrial ROS content eventually resulted in a high vulnerability of lymphocytes, as opposed to the endurance of monocytes, against the stress induced by the flow cytometry procedure [39,40].

From the metabolic point of view, the global increase in mitochondrial ROS generation matched the increased oxygen consumption for tasks independent of ATP synthesis. This observation confirms the notion that, besides regulating energy asset, mitochondria contribute to promoting phenotype adjustment to the needed cell function, at least partially by modulating its redox balance [12,13,14]. A fundamental player in this adaptation is represented by the dynamic interactions between mitochondria and ER modulated by the MAMs [18,19,20].

The literature already widely reported that these membranes act as a crucial link for the exchange of molecular signals, protein complex formation and cellular response to alterations of its homeostasis [16] between different cell compartments. The present data confirm these concepts by documenting that the metabolic response to the mitochondrion-generated redox stress primarily involved the ER-PPP and its triggering enzyme H6PD as opposed to relatively less evident activation of the G6PD-triggered cytosolic PPP [20,41].

The response of ER metabolism to mitochondrial ROS generation is further corroborated by the increased uptake of 2-NBDG particularly evident in lymphocytes harvested from HL patients. Duplicating the model of ^18^F-fluoro-deoxyglucose uptake in PET imaging, the retention of this glucose analogue is usually considered as an index of overall glucose consumption [42,43,44]. Nevertheless, the increase in 2-NBDG fluorescence intensity, observed in HL PBMCs, was independent of glycolytic flux, directly estimated by ECAR. This piece of evidence agrees with several studies by our and other groups [24,25,26,27,33,45,46], suggesting a relevant role for ER-PPP in the uptake of de-oxygenated glucose analogues. Indeed, the univariate regression analysis reported that the increased 2-NBDG uptake in HL PBMCs was largely predicted by the disease effect on the redox stress (indexed by MDA content and MitoSOX signal) and the consequent ER metabolic response, leading to organelle extension and to the enhancement of H6PD catalytic function.

Obviously, the present data do not permit verifying the mechanistic role of ER-PPP activation in the development of the oxidative phenotype associated with the inflammatory activation. On one side, the reductive power of generated NADPH might improve the PBMCs’ endurance against the redox stress [47]. On the other side, it might provide the cofactor for NADPH-oxidases [48], as suggested by the evidence that the inhibitory effect of dehydroepiandrosterone on ROS generation implies the integrity of glucose-6P transport across the ER membrane [49]. Nevertheless, and whatever its role, the observed metabolic shift corroborates the pivotal role of ER and its connection with the mitochondria in the development of the oxidative phenotype of HL PBMCs.

From the methodological point of view, the high redox stress in the PBMCs of untreated HL patients might hamper the evaluation of chemotherapy side-effects. Usually, this task involves directly comparing metabolic or biological features of normal tissues and cells in cohorts encompassing control subjects and treated patients. This approach, however, cannot account for the direct interference of disease. Indeed, an evident redox stress has been already reported in children with lymphoma or leukemia even before the start of pharmacological treatment [50]. Accordingly, chemotherapy toxicity might most likely benefit from longitudinal studies evaluating the investigated variables both before and after treatment administration in the same patient.

Several limitations of the present study should be carefully considered. The limited number of HL patients obviously implies the need for further investigations to verify whether the divergent endurance of lymphocytes and monocytes against the redox stress contribute to the capability of their counts in peripheral blood to predict disease aggressiveness and response to chemotherapy. Nevertheless, it still allowed the identification of the main finding of the present study: the presence of oxidative stress in circulating lymphocytes and monocytes of HL patients already before chemotherapy toxicity.

As a further limitation, several variables (including enzymatic activity, MDA content, total antioxidant capacity, OCR and glycolytic flux) could not be separately investigated for the two PBMC populations because of the limited amount of available blood in these patients. This limitation prevented verifying the presence of possible differences in mitochondrial function between the studied lymphocytes and monocytes. Nevertheless, the divergent MitoSOX Red signal already indicates a greater respiratory uncoupling in the former as a possible contributor to their impairment.

A similar consideration also applies to the degree of mitochondrial damage. Indeed, the missing evaluation of OCR during OXPHOS uncoupling does not permit identifying a possible reduction in maximal mitochondrial respiratory capacity. Nevertheless, the significant decrease in ATP-linked mitochondrial OCR, coupled with the increased MitoSOX fluorescence, already confirms a primary role for these organelles in the oxidative damage of HL PBMCs.

The redox response to HL might be heterogeneous through the different subtypes of lymphocytes and monocytes populating the peripheral blood. This evaluation would have been feasible by combining the metabolic characterization with a precise definition of involved cell phenotype. Yet, this approach would have necessitated an extra blood sampling for CD staining that was avoided for ethical considerations in all subjects, except for the subset cohort involved in the described pilot test. Nevertheless, the present evidence of a significant metabolic shift induced by HL in PBMCs might represent the basis for future studies aiming to identify the involved cell subtypes.

Finally, the present study did not investigate the molecular mechanisms underlying the observed oxidative damage in the PBMCs of untreated HL patients. This task would have implied seeding and culturing these cells in media whose content of nutrients and signals cannot reproduce the peripheral blood environment. Obviously, this difference might per se alter both ROS generation and antioxidant reaction. On the other hand, the observed compartmentalization of redox stress within mitochondria and ER lumen provides a new insight into this metabolic alteration, suggesting the need for new hypotheses about the involved pathways and their potential role in HL aggressiveness.

## 5. Conclusions

Our results indicate oxidative damage in the PBMCs of HL patients before the beginning of therapy. The redox stress reflects a relative mitochondrial OXPHOS uncoupling that primarily extends to the ER, whose homeostasis is strictly dependent upon the activity of the local PPP. This finding configures the ER–mitochondria connection as a fundamental player in the host response to disease. This direct HL effect should be carefully considered to better understand the mechanisms underlying chemotherapy toxicity in normal tissues.

## Figures and Tables

**Table 1:** Demographic and hematologic data.

	Control Subjects	*p* Value	HL
**N**	62		19
**Nr of female (%)**	30 (48%)	ns	7 (37%)
**Age**	48 ± 17	ns	44 ± 17
**RBC (millions per µL)**	4.77 ± 0.48	ns	4.68 ± 0.40
**HB (g/L)**	140.03 ± 17.73	ns	131.81 ± 15.85
**HT (%)**	41.39 ± 4.69	0.041	37.39 ± 9.52
**PLT (thousands per µL)**	234.74 ± 51.93	0.003	305.31 ± 117.74
**WBC (thousands per µL)**	6.08 ± 1.51	0.000	9.13 ± 3.72
**Neutrophils (thousands per µL)**	3.34 ± 1.20	0.000	6.46 ± 3.76
**Lymphocytes (thousands per µL)**	1.94 ± 0.63	ns	1.70 ± 0.73
**Monocytes (thousands per µL)**	0.47 ± 0.14	0.000	0.77 ± 0.35

HL: Hodgkin’s Lymphoma; RBC: Red blood cell count; HB: Hemoglobin; HT: Hematocrit; PLT: Platelet count; WBC: White blood cell count.

**Table 2:** Prediction of 2-NBDG uptake by univariate regression analysis.

	Unstandardized Coefficients		Standardized Coefficients		
	B	Std. Error	Beta	t	*p* Value
Basaline OCR	0.012	0.056	0.027	0.217	0.829
Gender Male (1) Female (0)	2.11	9.309	0.027	0.227	0.821
Basaline ECAR	−0.081	0.105	−0.097	−0.77	0.444
Maximal ECAR	−0.09	0.097	−0.116	−0.929	0.356
ATP-linked OCR (% of control)	−174.828	89.337	−0.451	−1.957	0.069
ATP-independent OCR (% of control)	179.588	87.229	0.469	2.059	0.057
Age (years)	0.56	0.288	0.225	1.949	0.055
**MDA**	**1.896**	**0.927**	**0.251**	**2.045**	**0.045**
**G6PD (mU/mg)**	**0.262**	**0.119**	**0.265**	**2.199**	**0.031**
**HL (1) Control (0)**	**40.117**	**9.506**	**0.445**	**4.22**	**0.000**
**Viable FCM PBMC (%)**	**−1.521**	**0.245**	**−0.596**	**−6.21**	**0.000**
**H6PD (mU/mg)**	**21.488**	**4.216**	**0.777**	**5.096**	**0.000**
**ERT MEAN MFI**	**0.803**	**0.073**	**0.794**	**10.935**	**0.000**
**MitoSOX MEAN MFI**	**0.193**	**0.016**	**0.82**	**11.983**	**0.000**

HL: Hodgkin’s lymphoma; OCR: oxygen consumption rate; ECAR: extracellular acidification rate; MFI: mean fluorescence intensity; MDA: malondialdheyde; FCM PBMC: flow cytometric PBMCs; G6PD: glucose-6-phosphate-dehydrogenase; H6PD: hexose-6-phosphate-dehydrogenase; ERT: ERTracker.

## Data Availability

Data is contained within the article.
